# Interlocking Evaluation of Mesoscopic Skeleton with the Compaction Degree of Hot-Mix Asphalt

**DOI:** 10.3390/ma16175879

**Published:** 2023-08-28

**Authors:** Xiangbing Gong, Ziming Liu, Guoping Qian, Zhiyang Liu

**Affiliations:** 1 School of Transportation Engineering, Changsha University of Science and Technology, Changsha 410114, China; 2 College of Civil and Transportation Engineering, Shenzhen University, Shenzhen 518060, China

**Keywords:** asphalt mixture, digital image processing technology, fine-grained scale, 3D refactoring, skeleton structure

## Abstract

Asphalt mixtures are multi-phase composites composed of aggregates, bitumen, mineral powders, and voids, and various structures are intertwined during the compaction process. Most of the traditional research focuses on the macro-scale domain, and it is difficult to obtain the internal structure of asphalt mixture in different compaction processes. With the continuous development of digital image technology, the influence of the meso-structure of the asphalt mixture on the compaction quality of the asphalt mixture has become a new means to evaluate the performance of the asphalt mixture. In this paper, different numbers of compactions are selected to represent different stages in the compaction process, the digital images of specimens in different compaction stages are obtained by industrial CT scanning technology. Then, the images are processed and reconstructed in three dimensions using improved image segmentation methods, and the position characteristics and geometric information of coarse aggregate are obtained by combining the Oriented Bounding Box (OBB). The meso-response characteristics of the skeleton structure of the asphalt mixture during compaction were studied. The influence of the internal structure of the mixture on the compaction quality of the mixture was obtained, which is of great significance for the study of improving the durability of the pavement. The results show that the “effective coordination number” (the number of aggregate particles that can transmit force in the skeleton structure) is greatly related to the aggregate size. With the compaction process, the centroid of coarse aggregate in the upper layer of the specimen reflects the overall downward movement trend. The inclination angle of the aggregate spindle tends to be in the range of 80°~100°; the anisotropic amplitude of the xy plane increases, and the direction of the aggregate spindle becomes more and more consistent. With the increase in the number of rotational compactions, these four parameters showed obvious rules, indicating that this meso-characteristic index could well characterize the compaction quality of the asphalt mixture in the compaction process.

## 1. Introduction

The durability of the road is not only closely related to the asphalt mixture material factors, structural factors also have a great impact on the durability of the road surface. And, the formation of asphalt pavement structure must be affected by the quality of compaction, so it is of great significance to study the change law of asphalt mixture structure during the compaction process. Nowadays, most of the mainstream evaluation indicators for compaction quality are macro volume characteristic indicators [1,2,3], which make it difficult to characterize the changes in the structure and the formation of the skeleton during the compaction process. Therefore, it may be more interesting to study the structural properties of the mixture compaction process under microscopic conditions.

Many studies often used macroscopic indicators such as compaction and porosity to evaluate compaction quality. Zhang evaluated compaction uniformity using density and porosity distribution and proved it feasible with a non-nuclear density meter PQI [1]. Pouranian proposed a theoretical analysis method to estimate the effect of different gradation aggregates on the skeleton structure of VMA (voids in mineral aggregate) and asphalt mixture by using the mixture accumulation model [2]. Chen proposed the rolling uniformity index (RUI) and rolling standard index (RSI) to control compaction quality [3]. Zhao used an intelligent algorithm to propose a new method for predicting the void ratio of the asphalt layer to evaluate compaction quality [4]. Zhao analyzed the radial angle of the aggregate in relation to compaction [5]. PEI tested compaction quality by detecting the voidness and uniformity of the mixture by GPR (ground penetrating radar) [6]. Dessouky proposed to use the working performance index and compaction performance index as the evaluation indicators of compaction [7]. CAI uses porosity, aggregate contact point, and aggregate inclination to evaluate the skeletal performance of the mixture after compaction [8].

Many experts and scholars have begun to apply digital image processing technology to the study of the mesoscopic structure of asphalt mixtures. The volume, distribution, shape, and direction of the asphalt mixture in the compaction process were obtained by digital image processing technology, which was used to study the skeleton of the asphalt mixture. Li used CT (computed tomography) to obtain the aggregate contact index (ACI) of aggregate contact characteristics and explored the effect of aggregate level pairing on DSAM (deformation stability of asphalt mixture) [9]. Airey used two-dimensional image acquisition and analysis technology, and the internal aggregate structure of asphalt mixture under different compaction methods was studied [10]. Guo compared the actual value of the coarse aggregate obtained by digital image processing technology with the equivalent sphere model and showed that the skeleton effect of the coarse aggregate gradually increases with compaction time [11]. Based on CT scanning and digital image processing technology, Xing verified the connection between the failure factor and the aggregate skeleton structure [12]. Onifade used image enhancement, image segmentation, and other techniques to process the digital images of asphalt concrete, and then studied the strength and deformation mechanism of the mixture’s mesostructure through finite element simulation [13]. Nejad used digital image processing technology to identify and detect the three phases (aggregate, glue, and void) of the asphalt mixture and compared it with the asphalt mixture compaction index measured in the laboratory. The results showed that the image processing algorithm can effectively evaluate the general performance and compaction quality of the mixture [14]. Masad proposed parameters to measure aggregate orientation, aggregate gradation, and air void distribution in AC (asphalt concrete) mixes, and used a computer automatic image analysis program to verify these parameters [15]. Sefidmazgi proposed a finer method to characterize the internal structure of aggregates to define performance-related parameters that can be used as quality indicators of the mixture [16]. Cristine analyzed asphalt mixtures compacted in the lab and in the field using digital imaging techniques to find the specimen molding method that is most representative of the field [17]. Huang used digital image processing (DIP) and image-pro plus 6.0 (IPP) software to obtain the principal axis orientation and interlayer distance as evaluation indicators to evaluate the migration characteristics of aggregate particles in asphalt mixtures [18]. Zhao reconstructed the three-dimensional microstructure of the FAM (fine aggregate mortar) specimen by microcomputer tomography (mCT) testing. Through microstructure analysis, a method for analyzing void quantity, volume, and space is proposed [19]. Alexis proposed a method for fabricating isolated FAM test specimens by using X-ray microcomputed tomography testing and digital image processing [20]. Coleri used X-ray computed tomography images taken before and after the rut test to investigate changes in the microstructure of asphalt concrete caused by full-scale accelerated pavement tests [21]. Coenen, Masad, and Nima Roohi Sefidmazgi used image processing to obtain the skeleton structure information of the aggregates in the mixture to study the compaction of the mixture [22,23,24]. Tashman used CT and image analysis software to study the void distribution of the mixture [25]. Zhu used the 3D blue light scanner to collect the morphological characteristics of aggregates and constructed a virtual aggregate database and an asphalt mixture model with aggregate morphological characteristics [26]. Liu used image processing technology to obtain the internal mesoscopic structure of the mixture for the study of the uniformity of the mixture [27]. Zelelew and Vadood used image processing technology to obtain aggregate and void parameters, which can be used to detect mixtures or build virtual models [28,29,30].

Many scholars have also studied the effect of asphalt mixture aggregate structure on compaction quality. Guarin evaluated the skeleton structure of aggregates using the concept of the dominant aggregate size range (DASR) and defined that DASR is a major player in the aggregate skeleton structure network [31]. CAI used the porosity and the number of aggregate contact points to evaluate the skeleton performance of the mixture, and the results showed that with the continuous incorporation of fine aggregates, the void ratio continued to decrease, and the number of aggregate contact points first increased and then decreased [8].

In summary, this paper studied the evolution of asphalt mixture skeleton structure in different compaction processes by using digital image processing technology. The Otsu segmentation method was improved to make it suitable for the segmentation of digital images of asphalt mixtures. Then, the three-dimensional reconstruction of the asphalt mixture was realized by Aviro 9.0 software, and the position characteristics and geometric information of the coarse aggregate were obtained by the Oriented Bounding Box (OBB). The mesoscopic skeleton structure characteristics of different compaction processes (rotational compaction 10, 16, 25, 40, 70)were calculated, the mesoscopic skeleton structure characteristics of different compaction processes were analyzed, and the evolution of asphalt mixture skeleton structure during the compaction process was explored.

## 2. Materials and Test Methods

### 2.1. Materials

The raw materials tested in this paper mainly include aggregates, mineral powder, and asphalt, and the selected materials should meet the specifications.

The asphalt binder used in this article is the SBS (styrene–butadienestyrene) modified asphalt produced by Maoming Lusheng Asphalt Co., Ltd. (Maoming, China), which is tested according to the “Highway Engineering Asphalt and Asphalt Mixture Test Regulations” (JTG E20-2011) [32], with a softening point of 58 °C, a ductility of 36 cm, and a penetration degree of 73, and its indicators meet the “Technical Specifications for Highway Asphalt Pavement Construction” (JTG F40-2004) [33].

The aggregates used in this article are all high-quality basalt, which are tested according to the “Highway Engineering Aggregate Test Procedures” (JTG E42-2005) [34], and the results are listed in the following Table 1 and Table 2.

The ore powder used in this paper is limestone mineral powder, which is tested according to the “Highway Engineering Aggregate Test Procedure” (JTG E42-2005) [34], and the experimental results meet the “Technical Specifications for Highway Asphalt Pavement Construction” (JTG F40-2004) [33].

### 2.2. Specimen Preparation for Different Compaction Processes

**Figure 1:**
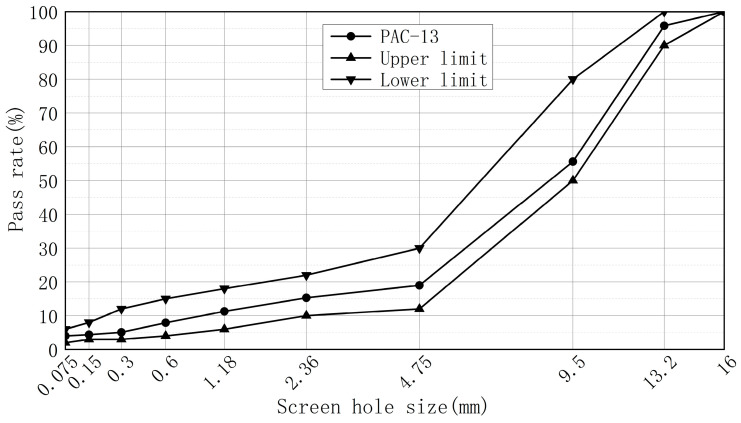
Synthesis of gradation curves.

This article uses the PAC-13 (drainage asphalt mixture with a maximum particle size of 13 mm) asphalt mixture, whose synthetic gradation curve is shown in Figure 1.

In this paper, the optimum asphalt content of the PAC-13 mixture is calculated based on the Technical Specification for Highway Asphalt Pavement Construction (JTG F40-2004) [33], and the optimum asphalt content of the PAC-13 mixture in this article is determined to be 4.10%.

The AASHTO specification developed by the American Association of State Highway and Transportation Officials specifies the relationship between the gyration compaction number and the amount of traffic. The gyration number (N-design) in this paper is chosen as 70 because the specimen height and porosity meet the requirements.

**Figure 2:**
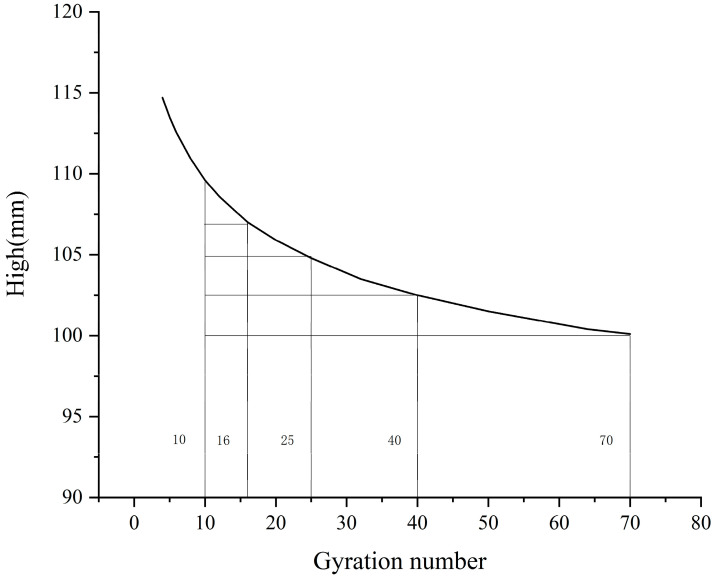
Height and gyration number diagram.

This paper studies the formation mechanism and evolution law of the asphalt mixture skeleton under different compaction processes, so it is required to select the appropriate gyration number as 0 to 70 times the standard for subsequent specimen molding. An SGC specimen with a height of 100 mm is formed using a gyratory compactor (TIPTOP, Changsha, China) and a plot of the height vs. gyration number is plotted, as shown in Figure 2.

The forming height of SGC specimens is 100 mm. As shown in the figure above, when the gyration number reaches 70 times, the specimen height is 100 mm, so 70 times is selected as the final gyration number; 10 is higher than the minimum gyration number specified in the AASHTO specification, and the specimen is not easy to loosen, which facilitates subsequent tests, so 10 times is selected as the initial gyration number. Between 10 and 70 rotation compactions, 16, 25, and 40 times are selected according to the principle of equal height difference of the specimen as the gyration number of the specimen during the compaction process. It was finally determined that the specimen used 10, 16, 25, 40, and 70 times as the gyration number.

This paper uses the rotary compaction test to form the asphalt mixture according to the test item T0702 mixed with JTG E20-2011 [32] “Highway Engineering Asphalt and Asphalt Mixture Test Regulations”, and it is proposed to make synchronous CT rotary compaction specimens for different compaction processes.

### 2.3. Digital Image Acquisition and Processing of Asphalt Mixtures

**Figure 3:**
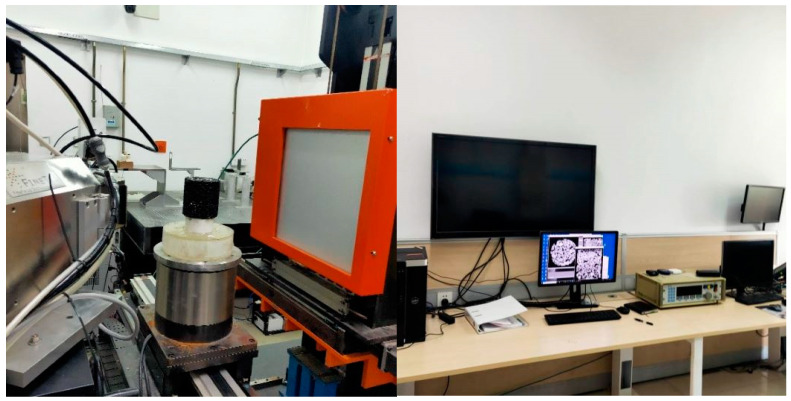
Industrial CT scan test instrument charts.

**Figure 4:**
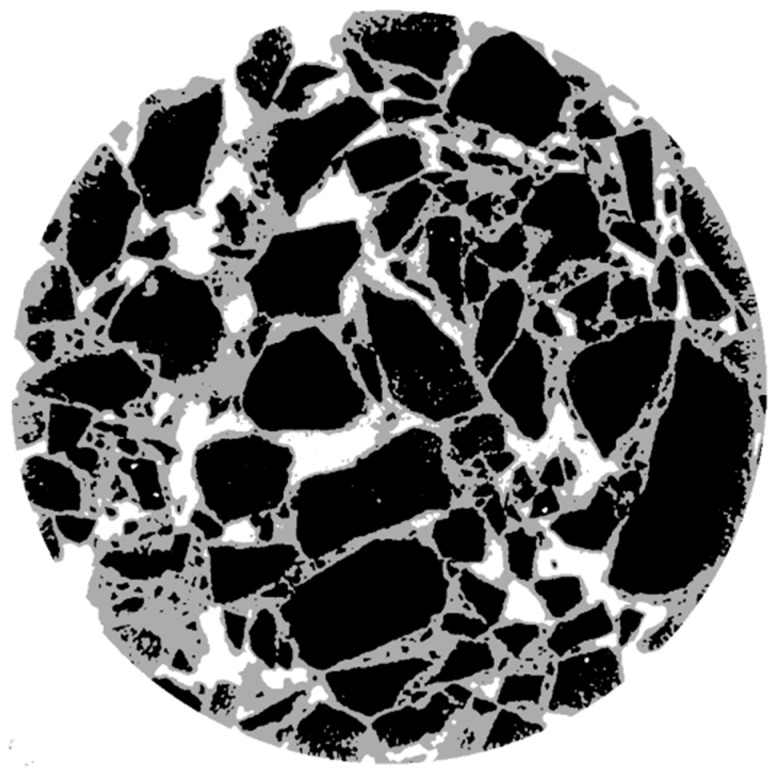
Effect drawing of improved Otsu segmentation.

The industrial CT scanning equipment used in this article is an industrial CT scanning 3D scanner (FineTec, Beijing, China) of the Capital Normal University, and the fluorescent tube model is FOMR 300.01Y. The test equipment is shown in Figure 3:

The use of industrial CT to obtain digital images of asphalt mixtures; unprocessed digital images are difficult to accurately analyze the content and distribution of each phase of the mixture, and it is also necessary to preprocess the CT pictures with the help of digital image processing technology. In this paper, the existing Otsu segmentation algorithm is improved, the parameters are continuously adjusted to adapt to the characteristics of the asphalt mixture, and the coarse aggregate, mortar, and gap three-phase structure are separated. Figure 4 shows the result of the segmentation using the above improved Otsu segmentation method.

**Figure 5:**
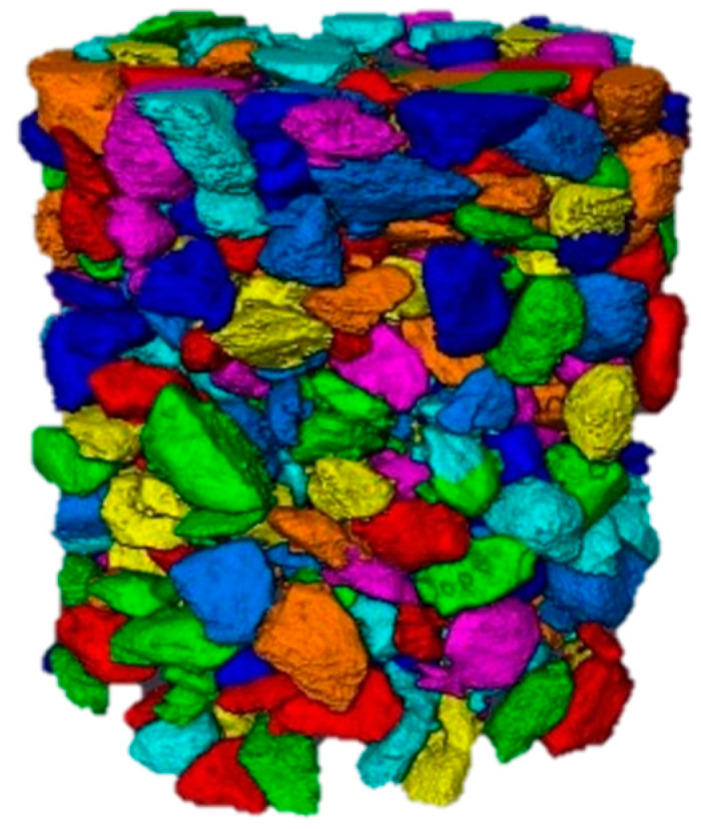
Minimum bounding box of all coarse aggregate.

It can be clearly seen from the segmentation effect of the above figure that the improved Otsu segmentation algorithm can clearly and accurately split the three-phase structure of aggregate, mortar, and void in the asphalt mixture. Therefore, the improved Ostu segmentation method is used to divide the asphalt mixture. Avizo 9.0 software is then used to achieve a three-dimensional reconstruction of the mesoscopic structure of the asphalt mixture. The result of the reconstruction is shown in Figure 5:

For the convenience of follow-up research, this paper divides the three-dimensional reconstructed coarse aggregate into three gears of 4.75 mm, 9.5 mm, and 13.2 mm according to the size of the sieve hole in the actual project. The three-dimensional reconstructed aggregate calculates its equivalent ball diameter according to the principle of volume equality, so as to determine its size gear. The formula for calculating the diameter of the equivalent ball is shown in the following equation:

$$D=\sqrt[{3}]{\frac{6V}{\pi }}$$

where *V* is the volume of the aggregate.

### 2.4. Parameters of the Aggregate

#### 2.4.1. Coordination Number

The coordination number is the most intuitive feature parameter of the rough aggregate embedded extrusion contact state at the mesoscopic scale, which can effectively describe the embedded extrusion contact state of the aggregate. For the asphalt mixture as a whole, the coordination number of a single aggregate is insufficient to characterize the embedded extrusion contact state and skeleton structure performance of the overall aggregate of the mixture, so the average coordination number is used to characterize the following:

(1) $$C=\sum_{i=1}^{N}\frac{P_{i}}{N}$$

where *P_i_* is coordination number of individual aggregate particles, *N* is the total number of aggregate particles, and *C* is the average coordination number.

At the same time, there will also be some aggregate particles with a coordination number of 0 and in the mixture as a whole, and these aggregate particles do not play a role in the mixed material skeleton structure and cannot play a role in transmitting forces and loads. Therefore, we can refer to these aggregate particles with coordination numbers of 0 and 1 as “invalid aggregate particles”. Correspondingly, the overall skeleton structural properties of the mixture can be characterized by the effective average coordination number as follows:

(2) $$C_{m}=\frac{\sum_{i=1}^{N}P_{i}-N_{1}}{N-N_{0}-N_{1}}$$

where *P_i_* is coordination number of individual aggregate particles, *N* is the total number of aggregate particles, *N*_0_ is the total number of aggregate particles with a coordination number of 0, *N*_1_ is the total number of aggregate particles with a coordination number of 1, and *C_m_* is the effective average coordination number.

#### 2.4.2. Centroid Distribution

In different compaction processes, it is possible to intuitively observe how the aggregate moves during the compaction process by distributing the centroids of each aggregate. In order to better quantify the distribution of the centroid in different compaction cases and more convenient analysis, it is stipulated that the standard rotary compaction specimen 1/3 and below (0~33.3 mm) is the lower layer, the standard rotary compaction specimen 1/3 to 2/3 (33.3~66.7 mm) is the middle layer, and the standard rotary compaction specimen 2/3 and above (66.7~100 mm) is the upper layer.

#### 2.4.3. Spindle Inclination

During the rotary compaction process, the structure of the asphalt mixture skeleton changes. The spindle inclination angle can describe the change of the arrangement and combination of skeleton particles in the rotational compaction process, so the spindle inclination angle is introduced to characterize the mesoscopic skeleton structure during the compaction process.

In this paper, 500 coarse aggregates with three-dimensional reconstruction are selected for statistics, and the aggregate particles of three-dimensional reconstruction are calculated by using the calculation method of the minimum enclosing box and obtain their spindle vectors, and the spindle vectors are used to calculate the inclination angle of the spindle on the three-dimensional space.

#### 2.4.4. Anisotropy of the Fabric Tensor

The fabric is used to describe the influence of geometric elements such as the arrangement of aggregate particles on its macroscopic mechanical properties under the mesoscopic scale domain, and the study of isomorphism has become a hot topic in recent years. The article by scholars such as Li Xuefeng [35] improved the calculation method of fabric tensor (the projection components of the three orthogonal planes are represented by using the long axis of the particles at the orthogonal plane projection angle, and the two-dimensional fabric tensor of each orthogonal plane is calculated). The method has sets of specimen material of asphalt mixture particles in the three orthogonal group on the specific amplitude of the fabric tensor, reflecting material fabric in the three orthogonal anisotropy of surface level. This calculation method is used in this article.

## 3. Experimental Results & Discussion

### 3.1. Coordination Number

**Figure 6:**
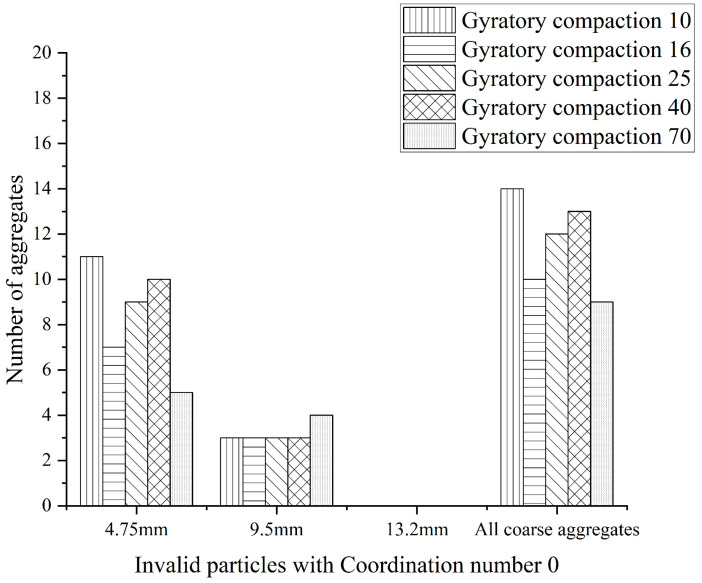
The coordination number of 0 “Invalid particle“.

**Figure 7:**
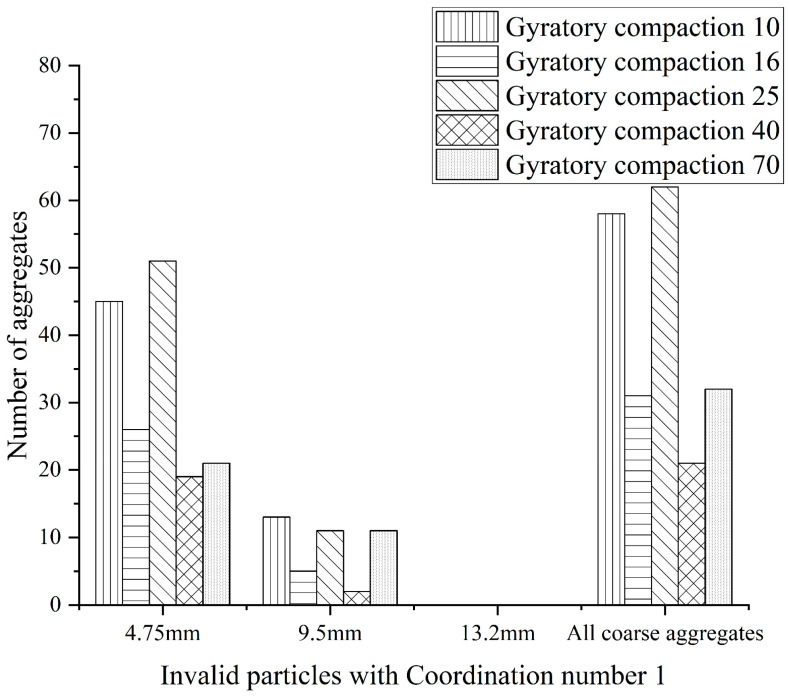
The coordination number of 1 “Invalid particle“.

A total of 500 coarse aggregates with three-dimensional reconstruction are selected for statistics, and the coordination number of test pieces with different compaction times is counted and calculated. The statistics and calculation results are shown in Figure 6 and Figure 7 below.

**Figure 8:**
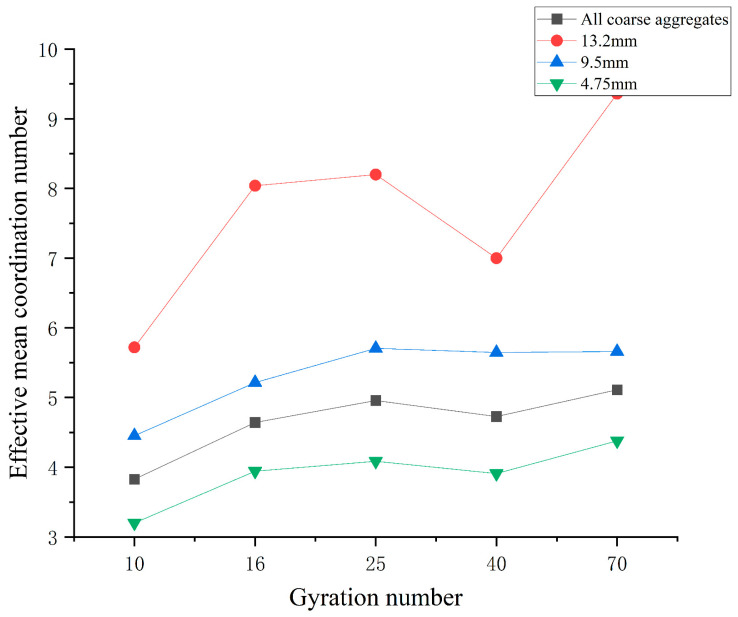
Compaction effective average number of different curves of the coordination number changes.

As can be seen from the effective average coordination number of Figure 8, the effective average coordination number of coarse aggregate in each group is 13.2 mm group > 9.5 mm group > 4.75 mm group, because the larger the aggregate size, the larger the surface area, the easier it is to contact with other coarse aggregates, and the larger the effective average coordination.

The effective average coordination number is generally an upward trend with the increase in the number of compactions, but in the period from 25 to 70 compactions, the coordination curve of the 13.2 mm gear has a significant downward and then upward trend. The above phenomenon may be caused by the increase in contact between aggregates under compaction 10 to 25 times, the increase in coordination numbers, and the formation of a sub-stable skeleton structure. In the stage of compaction 25 to 70 times, as the number of compactions increases, the original particle arrangement state is broken, the aggregate particles are rearranged, and the coordination number first decreases and then rises, forming a more stable skeleton structure.

### 3.2. Centroid Distribution

**Figure 9:**
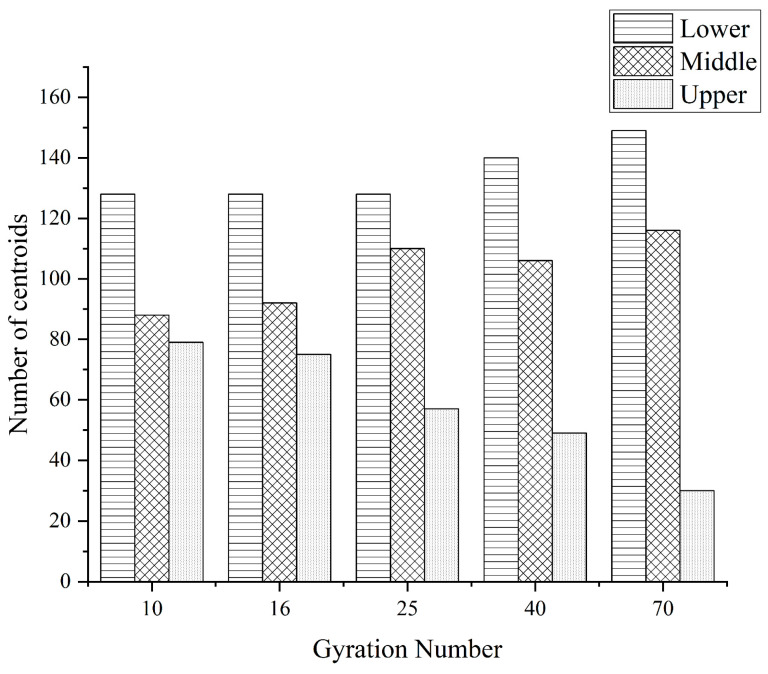
The 4.75 mm coarse aggregates under different stages of compaction of mass distribution.

**Figure 10:**
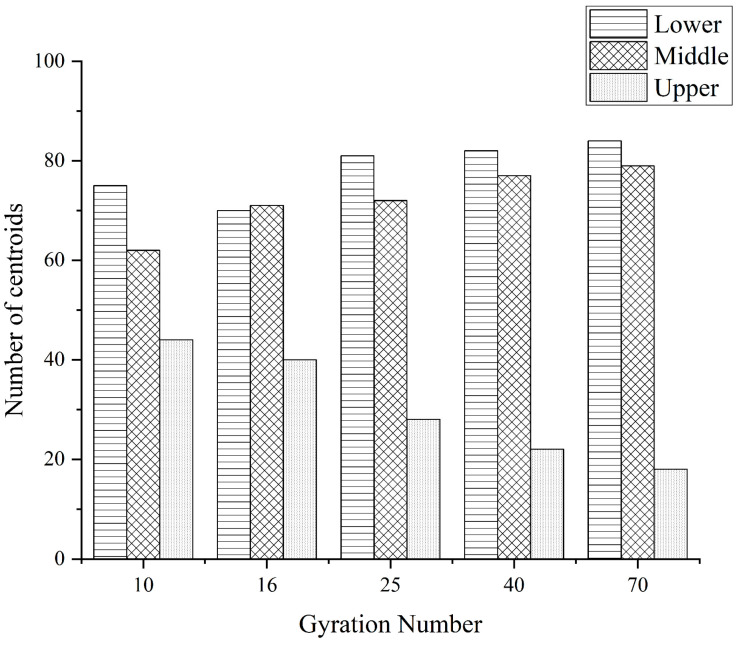
The 9.5 mm coarse aggregates under different stages of compaction of mass distribution.

**Figure 11:**
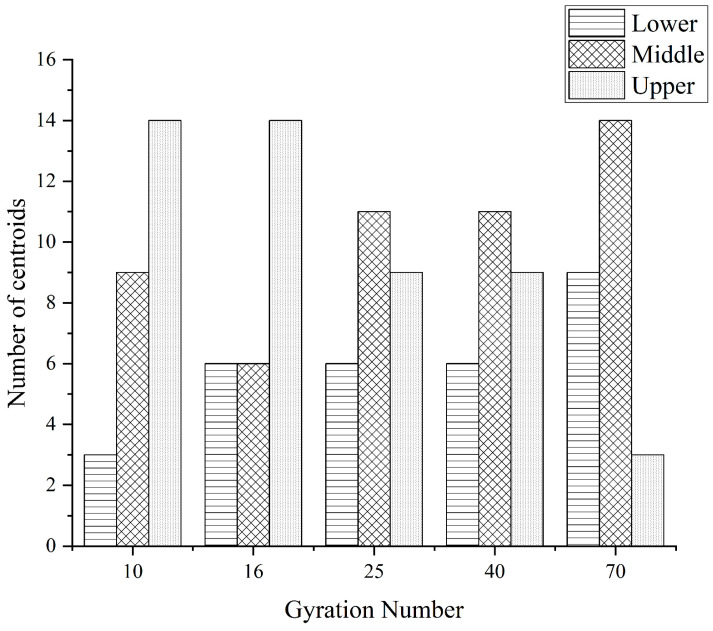
The 13.2 mm coarse aggregates under different stages of compaction of mass distribution.

In this paper, the distribution results of the core of coarse aggregates in the upper, middle, and lower layers under different compaction conditions are shown in Figure 9, Figure 10 and Figure 11 below.

It can be seen from the above figures that no matter what grade of coarse aggregate, with the increase of the number of compactions, the distribution of the centroid has a general similarity; with the increase of the number of compactions, the number of centroids in the upper layer begins to decrease and the numbers of centroids in the middle and lower layers begin to increase.

### 3.3. Spindle Inclination

**Figure 12:**
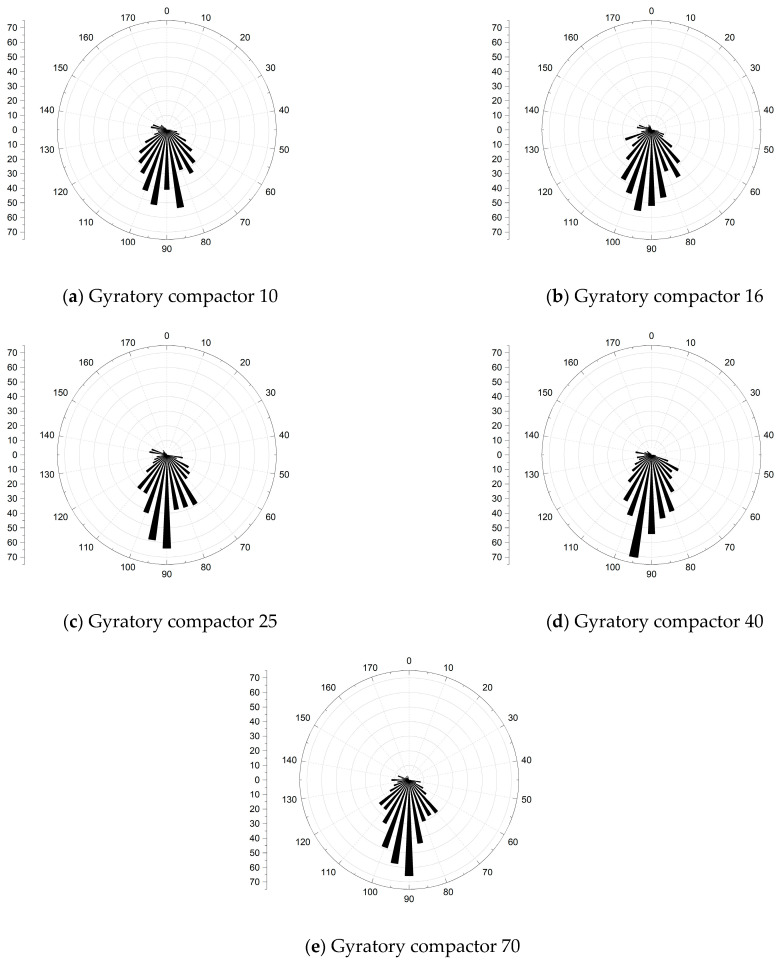
(**a**–**e**) Spindle inclination angle to wind rose diagram under different numbers of compactions.

In this paper, the aggregate spindle direction with the y direction as the positive direction is retained, so the value range of the spindle inclination angle is 0°~180°, and 5° is used as the interval length to calculate the change of the spindle inclination angle of different compaction processes. The statistical results are shown in Figure 12 below.

It can be seen from the spindle inclination wind direction rose chart that the distribution of the spindle inclination is symmetrically distributed at 90° as the symmetrical axis, and as the number of compactions increases, the spindle inclination of each aspect is also more and more concentrated near 90°. This shows that the spindle inclination angle from 80° to 100° is a stable angle, and as the number of compactions increases, more and more coarse aggregates are “lying flat”, and the skeleton structure is becoming more and more stable.

### 3.4. Anisotropy of the Fabric Tensor

**Figure 13:**
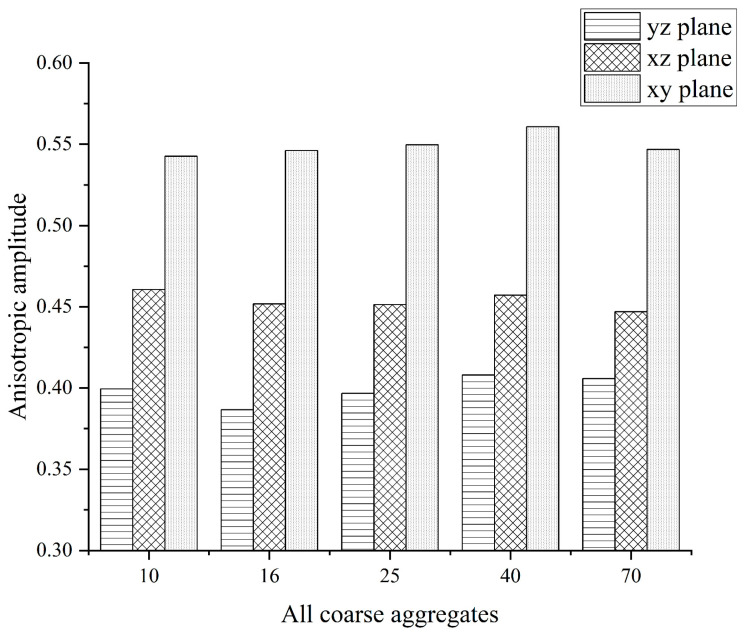
All coarse aggregates on a different amplitude of anisotropy of compaction case.

The opposite sex amplitudes of the specimens with 10, 16, 25, 40, and 70 rotation compactions were counted and calculated, respectively, and the results are shown in Figure 13.

As can be seen from the above figure, the anisotropic amplitudes on the xy plane are significantly greater than those in the xz plane and the yz plane, so the anisotropic amplitudes on the xy plane are selected in this paper to evaluate the anisotropy of the coarse aggregate configuration tensor of the rotating compaction specimen.

Before the rotation compaction 40 times, as the number of compactions increased, the anisotropic amplitude of the xy plane also increased, indicating that the arrangement of the coarse aggregate was more and more similar, and the spindle direction of the aggregate particles became more and more consistent. In the range of 40 rotational compactions to 70 rotational compactions, the anisotropic amplitude of the xy plane first decreased slightly, indicating that the arrangement and combination of aggregates were broken, and the aggregate particles were rearranged. Then, under the influence of compaction, the aggregate particles were rearranged in an orderly manner, the direction of the aggregate spindle became more and more consistent, and the anisotropic amplitude of the xy plane began to rise.

## 4. Conclusions

The traditional method uses compact degree to evaluate compaction quality, which is single and too macroscopic. This paper explains the formation law of the mixture from a mesoscopic perspective, which can better reflect the quality of compaction characteristics and make the evaluation index more advanced. In this paper, different number of compactions are selected to represent different stages in the compaction process. the mesoscopic structure of the specimens of different compaction processes is extracted by digital images. The mesoscopic structural characteristics such as the change of coarse aggregate coordination number, the centroid distribution of coarse aggregate, the Spindle inclination, and the anisotropy of the fabric tensor are studied and analyzed in different compaction processes. The formation law of mixture is explained from a mesoscopic point of view. The main conclusions are as follows:

(1) The “coordination number” of the specimen in different compaction processes is greatly related to the size of the aggregate, and the larger the aggregate size, the greater the possibility of producing “effective particles”. With the increase of the number of compaction, the “effective average coordination number” of each grade aggregate will increase accordingly, when the compaction number increases to 25~40 times, the skeleton structure of the asphalt mixture is relatively stable, the rearrangement phenomenon between the particles is not easy to occur, and the change of the “effective average coordination number” is relatively stable and the fluctuation is small.
(2) The distribution of centroids of different compaction processes of specimens has the following characteristics: with the increase of the number of compaction times, the number of centroids in the upper layer of the specimen continues to decrease, and the number of centroids in the middle and lower layers continues to increase.
(3) The frequency distribution curve of the spindle inclination angle of the specimen with different compaction times is 0°~180° and shows a Gaussian distribution state, especially in the range of 80°~100°, which is the most distributed. With the increase in the number of compactions, the standard deviation of the Gaussian distribution decreases, and the inclination angle of the aggregate spindle tends to be more and more in the range of 80° to 100°, indicating that there are more and more coarse aggregates for “lying flat”, and the skeleton structure is becoming more and more stable.
(4) In different compaction processes, the anisotropic amplitude of the fabric tensor of the xy plane is significantly greater than that of the xz plane and the yz plane. Before the rotational compaction 40 times, as the number of compactions increased, the amplitude of xy plane anisotropy increased significantly. After 40 compressions, the change tends to stop. It is stated that a compaction of 40 times is a critical number of compactions at which the skeleton structure of the mixture may have reached initial stability.

## Figures and Tables

**Table 1:** Coarse aggregate apparent density.

Aggregate specifications (mm)	16–13.2	13.2–9.5	9.5–4.75	4.75–2.36
apparent density (g/cm^3^)	2.774	2.758	2.792	2.772

**Table 2:** Fine Aggregate apparent density.

Aggregate specifications (mm)	2.38–1.18	1.18–0.6	0.6–0.3	0.3–0.15	0.15–0.075
apparent density (g/cm^3^)	2.643	2.655	2.725	2.631	2.522

## Data Availability

The data is not publicly available.
